# CCAAT/Enhancer binding protein β silencing mitigates glial activation and neurodegeneration in a rat model of Parkinson’s disease

**DOI:** 10.1038/s41598-017-13269-4

**Published:** 2017-10-19

**Authors:** Jose A. Morales-Garcia, Elena Gine, Elena Hernandez-Encinas, Diana Aguilar-Morante, Ana Sierra-Magro, Marina Sanz-SanCristobal, Sandra Alonso-Gil, Raul Sanchez-Lanzas, Jose G. Castaño, Angel Santos, Ana Perez-Castillo

**Affiliations:** 1 Instituto de Investigaciones Biomédicas (CSIC-UAM), Arturo Duperier, 4, 28029 Madrid, Spain; 2 Centro de Investigación Biomédica en Red sobre Enfermedades Neurodegenerativas (CIBERNED), 28031 Madrid, Spain; 3 Departamento de Biología Celular, Facultad de Medicina, UCM, Plaza Ramón y Cajal s/n, 28040 Madrid, Spain; 4 Departamento de Bioquímica Facultad de Medicina, Universidad Autónoma de Madrid, 28029 Madrid, Spain; 5 Departamento de Bioquímica y Biología Molecular, Facultad de Medicina, Universidad Complutense de Madrid, 28040 Madrid, Spain; 6 Present Address: Instituto de Biomedicina de Sevilla, IBiS, Hospital Universitario Virgen del Rocío/CSIC/Universidad de Sevilla. Departamento de Fisiología Médica y Biofísica, 41013 Sevilla, Spain

## Abstract

The CCAAT/Enhancer binding protein β (C/EBPβ) is a transcription factor involved in numerous physiological as well as pathological conditions in the brain. However, little is known regarding its possible role in neurodegenerative disorders. We have previously shown that C/EBPβ regulates the expression of genes involved in inflammatory processes and brain injury. Here, we have analyzed the effects of C/EBPβ interference in dopaminergic cell death and glial activation in the 6-hydroxydopamine model of Parkinson’s disease. Our results showed that lentivirus-mediated C/EBPβ deprivation conferred marked *in vitro* and *in vivo* neuroprotection of dopaminergic cells concomitant with a significant attenuation of the level of the inflammatory response and glial activation. Additionally, C/EBPβ interference diminished the induction of α-synuclein in the *substantia nigra pars compacta* of animals injected with 6-hydroxydopamine. Taking together, these results reveal an essential function for C/EBPβ in the pathways leading to inflammatory-mediated brain damage and suggest novel roles for C/EBPβ in neurodegenerative diseases, specifically in Parkinson’s disease, opening the door for new therapeutic interventions.

## Introduction

CCAAT/Enhancer Binding Protein β (C/EBPβ) is a member of the basic leucine zipper (bZIP) class of transcription factors. There are six members of this family, which exhibit a high sequence similarity in their C-terminal domain and a more diverse N-terminus^1,2^. C/EBPβ is expressed in numerous tissues, including liver, adipose tissue, kidney, lung, ovary, mammary gland, and hematopoietic tissues and participates in multiple cellular functions, including metabolism, cell proliferation and differentiation (depending on the cell context), tumorigenesis, and immune response^3–6^. This regulation takes place through the induction or repression of many genes involved in these processes, such as proliferative or differentiation related markers, cytokines, chemokines, and proinflammatory genes^7,8^. Consequently, due to its relevance in many cellular processes, C/EBPβ is also implicated in the pathogenesis of different diseases, e.g. cancer, bacterial infections, and inflammatory processes^9^.

Regarding the Central Nervous System (CNS) it has been shown that C/EBPβ mRNA is expressed in different areas of adult rodent brain^10,11^. C/EBPβ has been shown to play an important role in synaptic plasticity and memory formation, particularly in the hippocampus^12,13^, in neuronal differentiation^14,15^, and hippocampal neurogenesis^16^. More recently we, and others, have found that C/EBPβ regulates the expression of several genes implicated in inflammatory processes and brain injury^17–21^ and mice lacking C/EBPβ showed a reduced inflammatory response after kainic acid injection and exhibited a dramatic reduction in pyramidal cell loss in the CA1 and CA3 subfields of the hippocampus after kainic acid injection^22^. Interestingly, some authors have also suggested a possible link between C/EBPβ and neurodegenerative disorders^23–26^.

Parkinson disease (PD) is the second most prevalent neurodegenerative disease among the elderly, characterized by the loss of dopamine producing neurons (dopaminergic neurons) in a specific brain region, the ventral midbrain. This cell damage causes movement disabilities and several non-motor symptoms, such as sleep and cognitive problems. Age is a major risk factor for PD, although the precise molecular mechanisms underlying this disease are not fully understood. Then, a better understanding of the mechanisms underlying the development and progression of PD pathology is critical for finding new neuroprotective therapies. Several mechanisms have been implicated as critical to PD pathogenesis: oxidative stress, mitochondrial dysfunction, protein misfolding and aggregation, inflammation, glutamate excitotoxicity and apoptosis^27^. No specific process looks primary in all cases of sporadic PD, and those pathogenic mechanisms possibly act synergistically through complex interactions to promote neurodegeneration. As commented above, many studies during the last years support an important role of neuroinflammation in the pathophysiology of PD^28^. Indeed, activated glial cells have been detected in the *substantia nigra pars compacta* (*SNpc*) of patients concurrently with an increased expression of pro-inflammatory mediators^28^. In addition, different studies using preclinical models of PD indicate that inflammatory processes are instrumental in neuronal cell death^29,30^. Targeting the signaling pathways in glial cells responsible for neuroinflammation represents a promising new therapeutic approach designed to preserve dopaminergic neurons in PD patients, and therefore improving their quality of life.

The motor manifestations of PD can be successfully treated, for a limited period of time, with drugs that restore dopaminergic function. However, there is no treatment that forestalls the progressive degeneration of dopaminergic neurons, indicating that novel strategies to inhibit this neurodegeneration ought also be considered as potential therapeutics for PD in combination with the current treatment with levodopa. Given the important role of C/EBPβ in processes such as excitotoxicity and neuroinflammation, and the critical role of these processes in neurodegenerative disorders, including PD, we here sought to address the potential role of this transcription factor in different *in vitro* and *in vivo* models of PD. In the present study, C/EBPβ expression is shown to be increased in rats injected with the neurotoxin 6-hydroxydopamine (6-OHDA), indicating that elevated C/EBPβ expression levels might contribute to the development of this disease. To verify this hypothesis we silenced C/EBPβ gene in the *SNpc* of adult rats. C/EBPβ depletion in lesioned animals, results in a significant decrease of dopaminergic cell death, glial activation and α-synuclein protein expression levels. Collectively, our results suggest that C/EBPβ depletion could constitute a valuable new therapeutic intervention against PD.

## Results

### C/EBPβ expression increased after a 6-OHDA-induced dopaminergic damage *in vitro*

**Figure 1:**
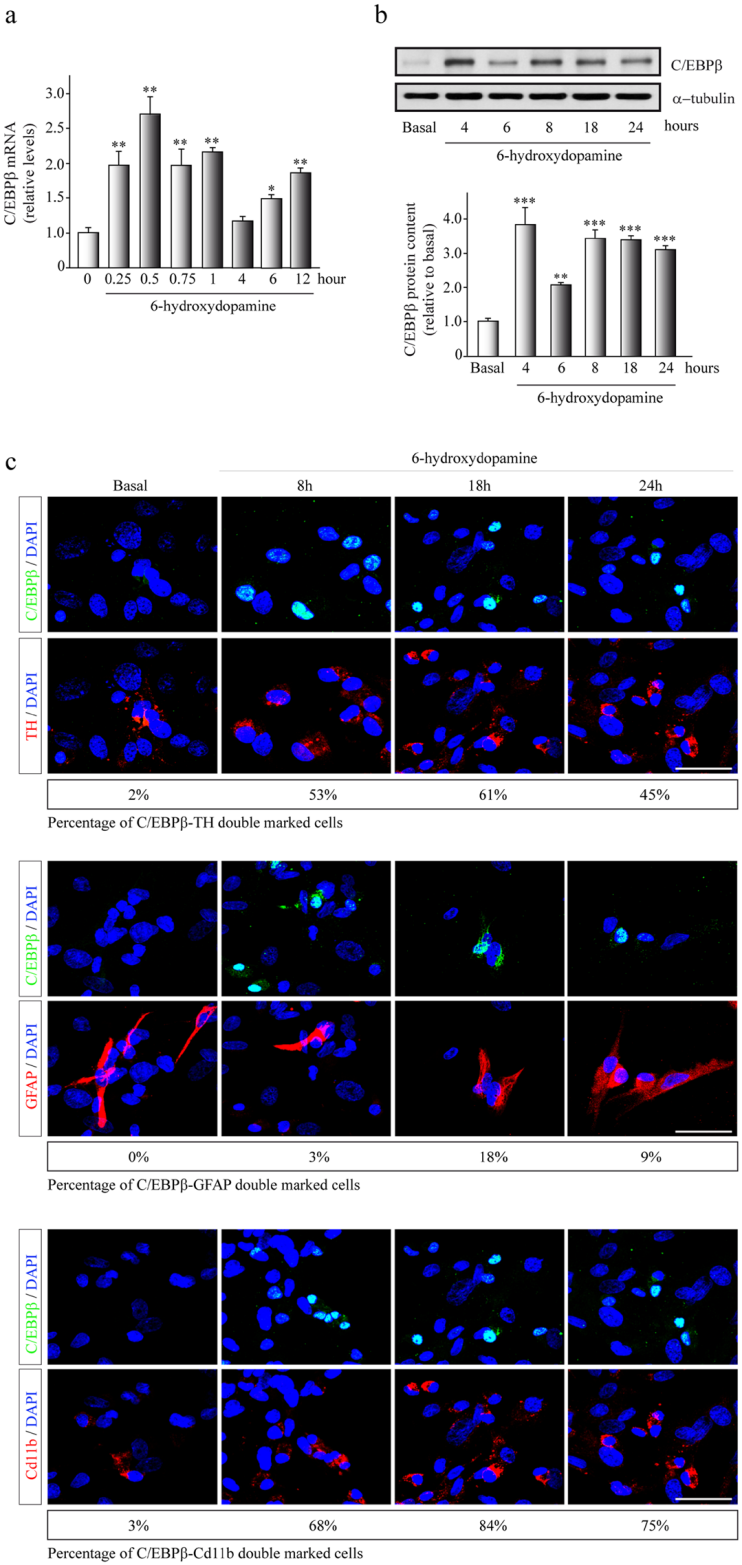
C/EBPβ expression on 6-OHDA-induced cell death. Dopaminergic cell line SH-SY5Y and rat primary embryonic mesencephalic cultures were treated with 6-OHDA (35 µM) for different times as indicated in Methods and several measurements were performed. (**a**) C/EBPβ mRNA levels analyzed after 6-OHDA-induced damage on SH-SY5Y cells. Data were obtained from ten independent experiments and presented as mean ± SD. *p ≤ 0.05; **p ≤ 0.01 versus non-treated (basal) cultures (**b**) Representative image of immunoblots and quantification showing C/EBPβ protein levels after damage on SH-SY5Y cells. Data were obtained from five independent experiments and presented as mean ± SD. **p ≤ 0.01; ***p ≤ 0.001 versus non-treated (basal) cultures. (**c**) Rat primary embryonic mesencephalic cultures were treated with 6-OHDA (35 µM) at different times and double immunofluorescence were performed. Images show co-expression of C/EBPβ (green) together in red with tyrosine hydroxylase (TH), a dopaminergic marker; glial fibrillary acidic protein (GFAP) for astroglial cells or Cd11b to label activated microglia. Representative results of three independent experiments are shown. The percentage of C/EBPβ double stained cells together with TH, GFAP or Cd11b is shown. Scale bar, 20 µm. Nuclei were counterstained with DAPI.

We have previously shown that C/EBPβ is overexpressed in neural cells after different insults^18,22^. Therefore, we first examined the expression levels of C/EBPβ in the dopaminergic cell line SH-SY5Y in response to treatment with the neurotoxin 6-OHDA. At the indicated times after 6-OHDA treatment, mRNA (qReal-time PCR) and protein (Western blot analysis) revealed a significantly increased expression of C/EBPβ in these cultures (Fig. 1a,b, ANOVA, *F* (7, 26) = 6.816, *p* < 0.0001 and F (5, 12) = 365.751, *p* < 0.0001 res*p*ectively). In basal conditions, a low expression of *c/ebpβ* mRNA was detected. In response to 6-OHDA a rapid induction of this transcription factor was observed within 0.25–0.5 h after damage followed by a decrease at 4 h and a new rise, which persisted for 6–12 h.

Next, we analyzed the protein levels of C/EBPβ in primary embryonic mesencephalic cultures, an *in vitro* model closer to physiological conditions (Fig. 1c). Cultures were incubated during 8, 18 and 24 hours in the presence of 6-OHDA, and two parameters were evaluated by fluorescent immunocytochemistry: the presence of C/EBPβ and its co-expression in the different cellular types in culture. As shown in Fig. 1c a marked induction of C/EBPβ as a results of 6-OHDA exposure was observed in dopaminergic neurons and microglial cells at all times analyzed. Also, to a lesser extent, an induction in C/EBPβ expression levels was detected in astrocytes.

### C/EBPβ depletion on SH-SY5Y resulted in an attenuation of inflammation after 6-OHDA injury

To further evaluate the possible effect of C/EBPβ gene silencing in the inflammatory response elicited by 6-OHDA, we infected SH-SY5Y cultures with lentiviral particles containing different sequences targeting C/EBPβ. Based on the highest effectiveness of interference obtained with the different sequences, we finally performed *in vitro* experiments using clones FC1 (containing a shRNA specific for C/EBPβ) and FS (containing a Non-Targeting shRNA), as described in Methods.

**Figure 2:**
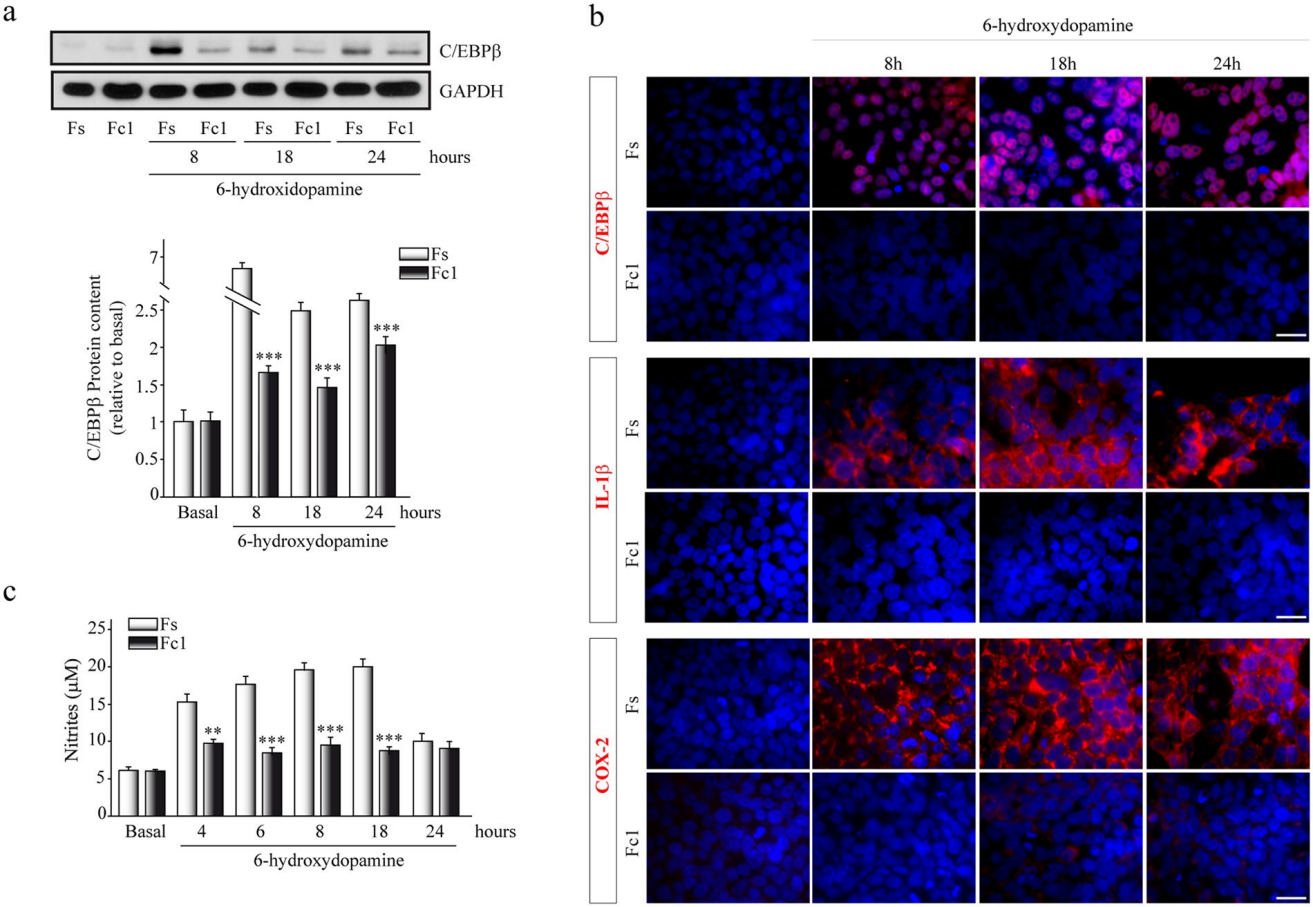
Gene silencing of C/EBPβ has an anti-inflammatory effect in SH-SY5Y cells. Cells were infected with lentiviral particles containing a Non-Targeting shRNA (control, Fs) or with shRNA targeting C/EBPβ (Fc1). Cultures were treated with 6-OHDA (35 µM) for different times, as indicated. (**a**) Representative immunoblots and quantification showing C/EBPβ protein levels in Fs and Fc1 cultures after damage at indicated times. Data were obtained from five independent experiments and presented as mean ± SD. ***p ≤ 0.001 versus non-treated (basal) cultures. (**b**) Representative images showing C/EBPβ, IL1β and ciclooxigenase-2 (COX-2) expression determined by immunofluorescence. Scale bar, 25 µm. (**c**) Nitrite concentration determination. Each data point represents the mean ± SD of 6 replications in 3 different experiments. **p ≤ 0.01; ***p ≤ 0.001 versus control (Non-Targeting shRNA) cultures.

As shown in Fig. 2a infection of SH-SY5Y cells with sh-C/EBPβ (FC1 clone) led to a drastic reduction of C/EBPβ expression in comparison with FS clones, after exposure to 6-OHDA.

The immunocytochemistry analysis displayed in Fig. 2b corroborates these results showing that the protein expression levels of C/EBPβ are clearly down-regulated in those cells expressing a shRNA specific for this protein (FC1 cells), at all the times studied. In parallel with this reduction, we also detected a clear decrease in the levels of two pro-inflammatory agents: COX-2 and IL-1β (Fig. 2b), as well as in nitrites liberation (Fig. 2c, ANOVA, *F* (11, 348) = 3,708.23*, p* < 0.0001). Together, these results suggested that C/EBPβ might help to modulate the increase in inflammatory mediators induced by 6-OHDA.

### C/EBPβ expression increased in the *Substantia nigra pars compacta* in the 6-OHDA rat model of Parkinson disease

**Figure 3:**
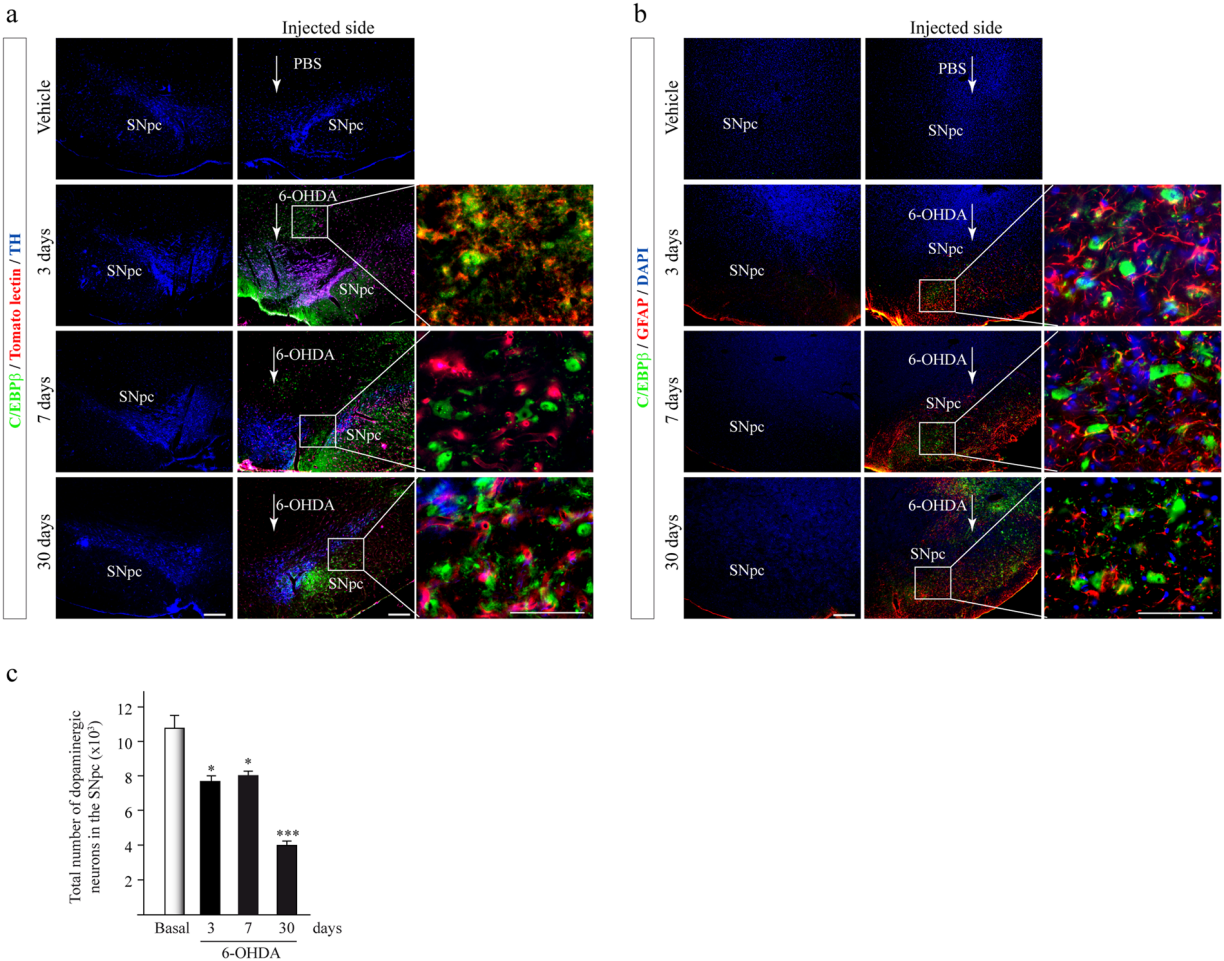
Injection of 6-OHDA in the Substantia nigra pars compacta (SNpc) of adult rats significantly induces C/EBPβ overexpression in a *in vivo* model of Parkinson’s disease. Rats were injected into the SNpc with 6-OHDA (5 μg). After 3, 7, and 30 days animals were sacrificed, and the brains processed for immunofluorescence, as indicated in Methods. (**a**) Triple immunofluorescence representative images of both hemispheres (contra- and ipsilateral) showing C/EBPβ expression (green) together with tomato lectin (red) an activated microglia marker and tyrosine hydroxylase (TH, blue). (**b**) Immunofluorescence images of both hemispheres (contra- and ipsilateral) showing C/EBPβ expression (green) together with GFAP (red) to label astroglial cells. Insets show high magnification of the selected area. Nuclei were stained with DAPI. Scale bar = 100 μm. (**c**) Quantification of the total number of dopaminergic neurons in the SNpc is shown. ***p ≤ 0.001, *p ≤ 0.05 versus control (PBS-injected animals).

We next investigated the *in vivo* role of C/EBPβ in a clinically relevant model of PD. Adult rats were injected in the *SNpc* with either saline or 6-OHDA and later on sacrificed 3, 7 or 30 days post-injection. Brain tissue was prepared for immunohistochemical analyses. The results shown in Fig. 3 revealed that the injection of 6-OHDA noticeably increased the expression of C/EBPβ on the ipsilateral injected side of the *SNpc* in rats when compared with saline-injected controls 3, 7 and 30 days after injection. C/EBPβ expression was mainly detected in the damaged area of the *SNpc*. This increase was more pronounced starting from 7 days post-injection. To ascertain a possible colocalization of C/EBPβ with the different cellular types in the *SNpc*, multiple labeling was performed using tomato lectin, anti-GFAP, and anti-TH to identify microglia, astrocytes, and dopaminergic neurons, respectively. As depicted in Fig. 3, C/EBPβ was detected in the nuclei of many microglial and astroglial cells and in those dopaminergic cells remaining in *SNpc* at 3, 7 and 30 days after the injection of 6-OHDA. These data are in agreement with those obtained *in vitro*, showing an induction on the expression of C/EBPβ in glial and dopaminergic cells.

### C/EBPβ silencing in the *Substantia nigra pars compacta* notably reduced dopaminergic cell death in hemiparkinsonian rats

**Figure 4:**
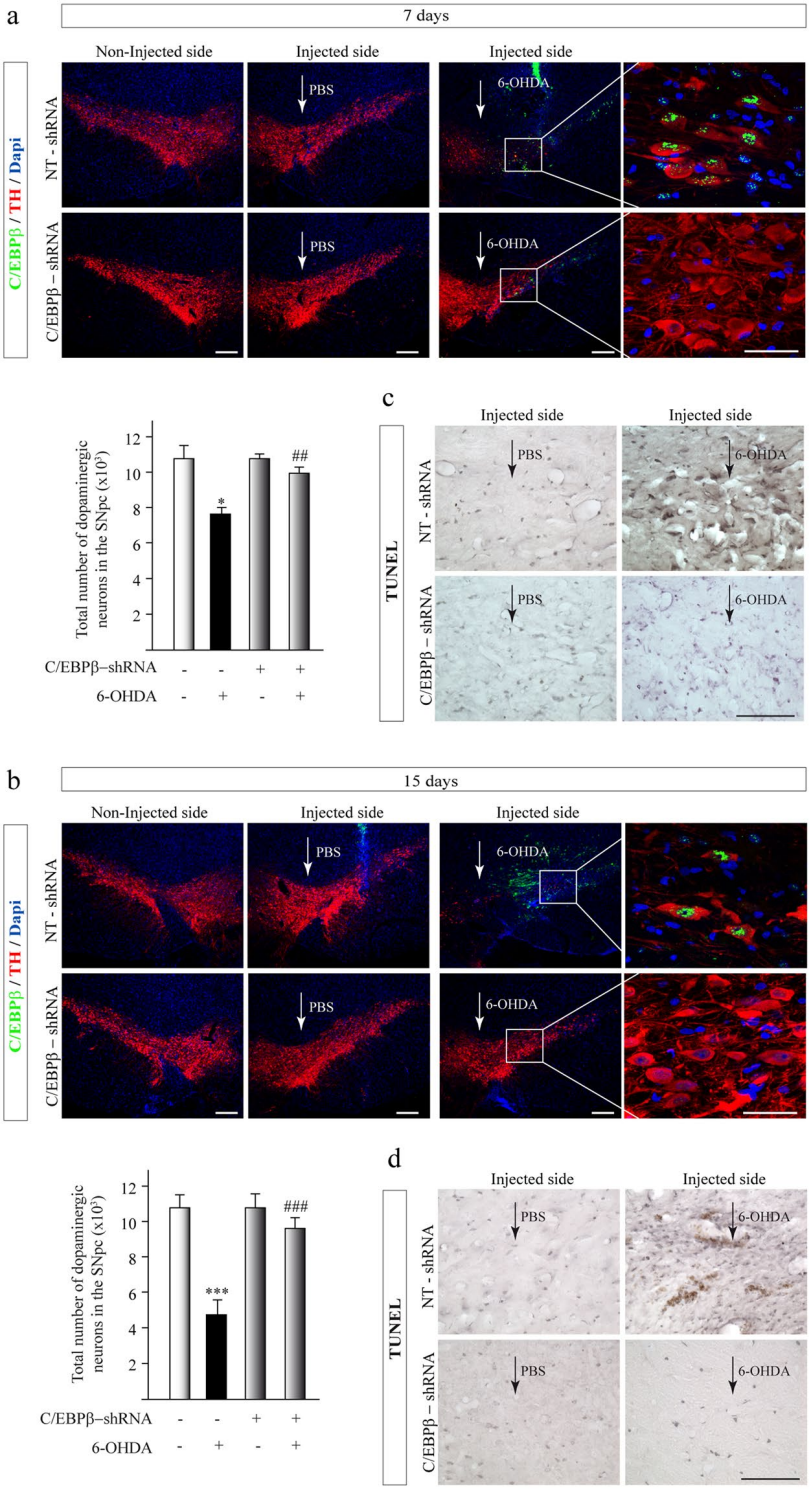
Injection of lentiviral particles containing C/EBPβ shRNA in the Substantia nigra pars compacta of adult rats significantly reduces dopaminergic neurodegeneration in a *in vivo* model of Parkinson’s disease. Rats were injected into the SNpc with lentiviral particles containing Non-Targeting shRNA (NT-shRNA) or C/EBPβ-shRNA in combination or not with 6-OHDA (5 μg). After 7 (**a**,**c**) and 15 (**b**,**d**) days animals were sacrificed, and the brains processed for immunofluorescence, as indicated in Methods. (**a**), (**b**) Double immunofluorescence representative images of both hemispheres (contra- and ipsilateral) showing C/EBPβ expression (green) together with the dopaminergic marker tyrosine hydroxylase (TH, red). Nuclei were stained with DAPI. Insets show high magnification of the selected area. Scale bar = 100 μm. Quantification of the total number of dopaminergic neurons in the SNpc is shown. ***p ≤ 0.001 versus control (NT-shRNA animals injected with PBS); ^###^p ≤ 0.01; versus NT-shRNA animals injected with 6-OHDA. (**c**,**d**) Coronal sections containing the SNpc were processed for TUNEL-assay.

In view of the results obtained in the *in vitro* and *in vivo* models of PD, indicating an induction in C/EBPβ levels after an injury with 6-OHDA, the role of C/EBPβ gene silencing on dopaminergic cells death in hemiparkinsonian rats was examined. To this end, rats were injected into the *SNpc* with lentiviral particles containing a non-targeting shRNA (NT-shRNA, control) or C/EBPβ-shRNA in combination with 6-OHDA or saline, and cell death was evaluated 7 and 15 days after injury (Fig. 4). As expected, quantification of the total number of dopaminergic cells in the *SNpc* showed a substantial loss of dopaminergic cells in the *SNpc* was observed 7 and 15 days after 6-OHDA injection. In contrast, those animals injected with lentiviral particles containing a specific shRNA for C/EBPβ together with this neurotoxin did not present a significant decrease of dopaminergic cells, indeed indicating that C/EBPβ gene silencing had a significant neuroprotective effect of dopaminergic cells in this area of the brain (Fig. 4a,b). Next, we analyzed the extent of apoptosis determining the number of TUNEL-positive cells in the *SNpc* 7 and 15 days after injection of 6-OHDA in rats. Results in Fig. 4(c,d) showed that 6-OHDA injection resulted in a significant increase in the number of TUNEL-positive cells in the *SNpc*, respect to controls (PBS-injected) animals. On the contrary, RNAi-mediated inhibition of C/EBPβ expression in the *SNpc* led to substantial cellular protection, and no significant differences were observed between control non-lesioned animals and 6-OHDA-lesioned animals injected with C/EBPβ-shRNA lentiviral particles, at both times analyzed. These results suggested that, at least in part, the degeneration of dopaminergic cells occurs through an apoptotic process and that the depletion of C/EBPβ also reversed this process.

### Intranigral delivery of C/EBPβ lentivirus particles significantly reduces 6-OHDA-induced glial activation in hemiparkinsonian rats

**Figure 5:**
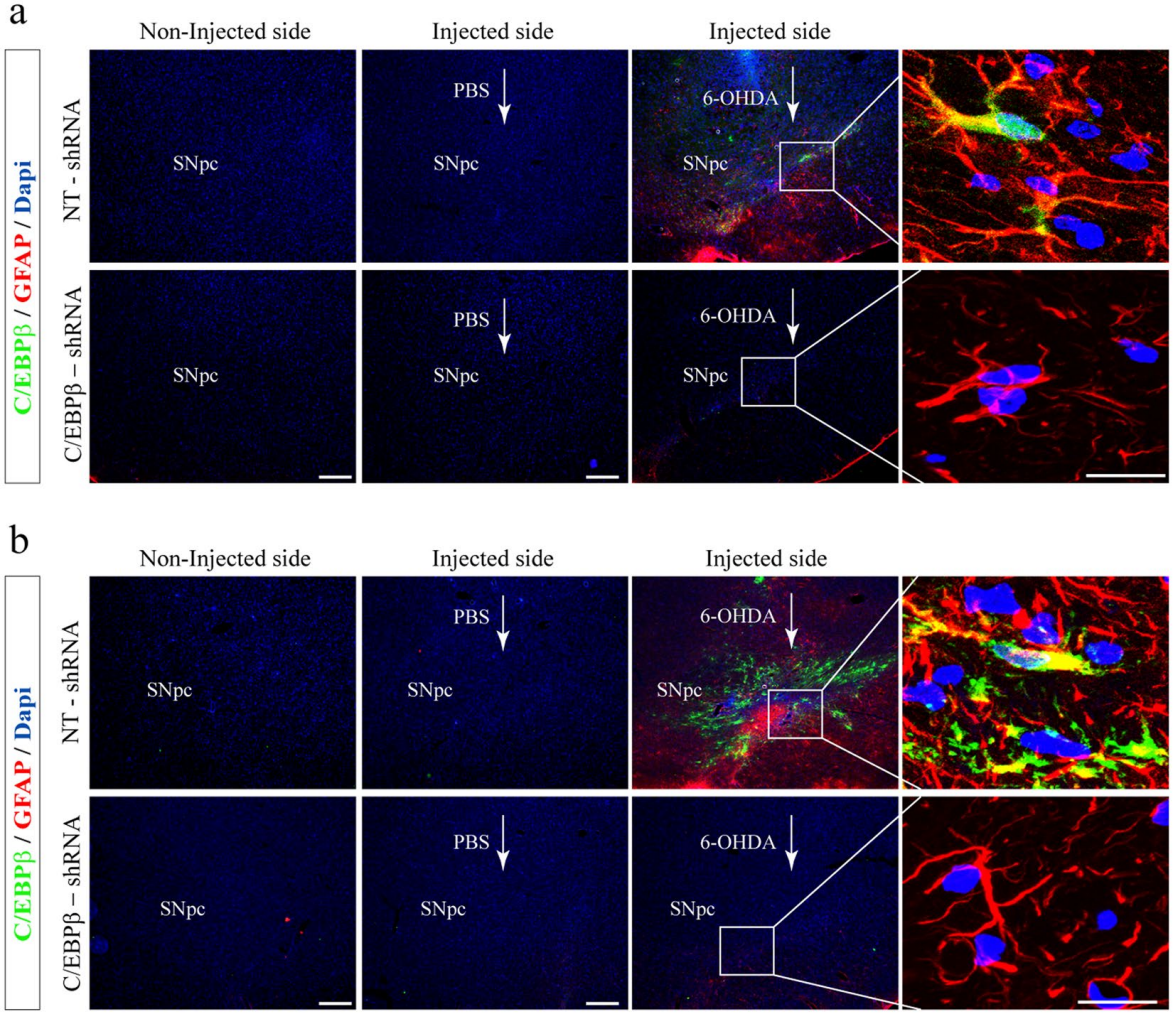
Injection of lentiviral particles containing C/EBPβ shRNA in the Substantia nigra pars compacta of adult rats significantly reduces astroglial activation in a *in vivo* model of Parkinson’s disease. Rats were injected into the SNpc with lentiviral particles containing Non-Targeting shRNA (NT-shRNA) or C/EBPβ-shRNA in combination or not with 6-OHDA (5 μg). After 7 (**a**) and 15 (**b**) days animals were sacrificed, and the brains processed for immunofluorescence, as indicated in Methods. Double immunofluorescence representative images of both hemispheres (contra- and ipsilateral) showing C/EBPβ expression (green) together with the astroglial marker glial fibrillary acidic protein (GFAP, red). Nuclei were stained with DAPI. Insets show high magnification of the selected area. Scale bar = 100 μm.

**Figure 6:**
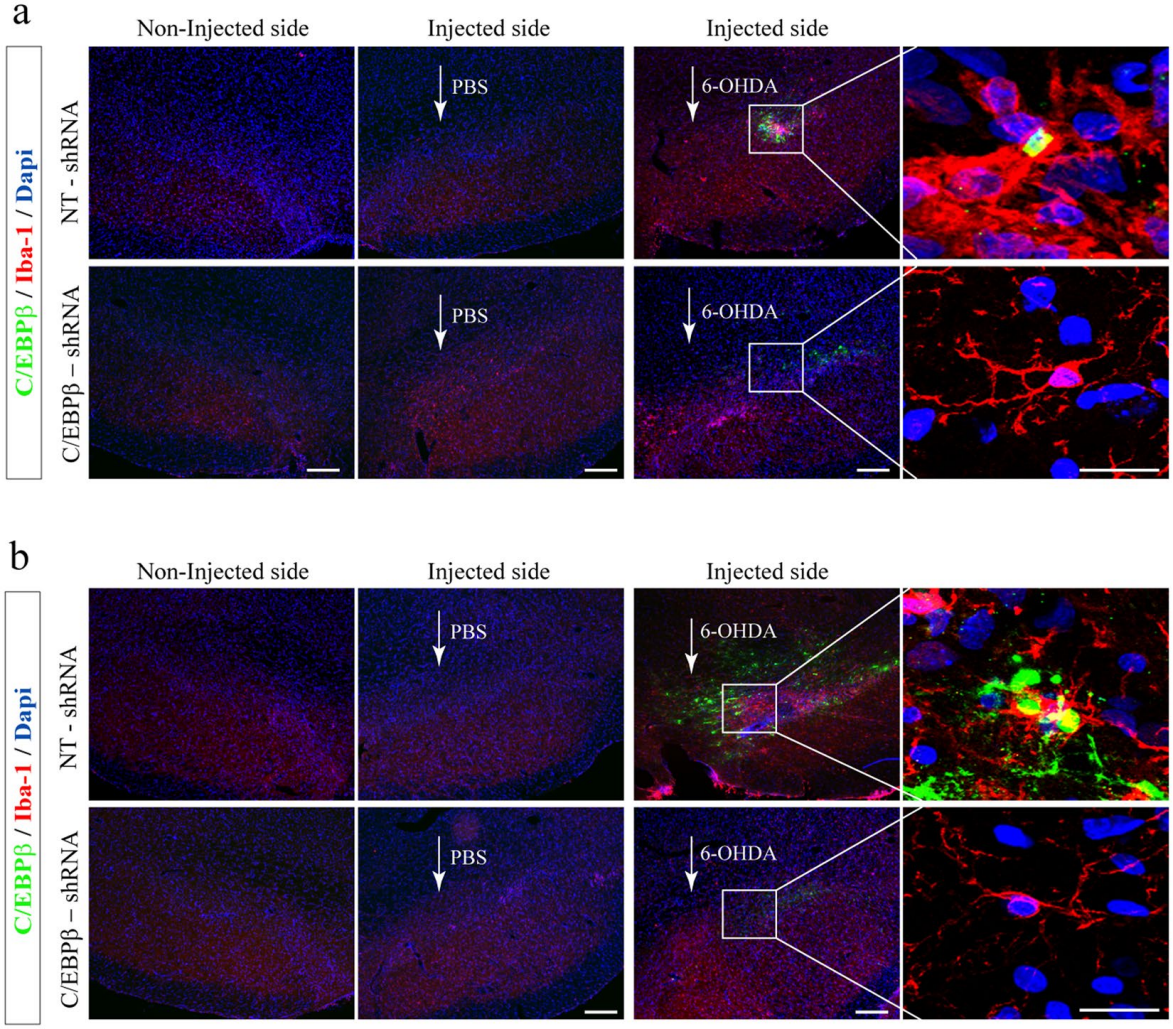
Injection of lentiviral particles containing C/EBPβ shRNA in the Substantia nigra pars compacta of adult rats significantly reduces microglial activation in a *in vivo* model of Parkinson’s disease. Rats were injected into the SNpc with lentiviral particles containing Non-Targeting shRNA (NT-shRNA) or C/EBPβ-shRNA in combination or not with 6-OHDA (5 μg). After 7 (**a**) and 15 (**b**) days animals were sacrificed, and the brains processed for immunofluorescence, as indicated in Methods. Double immunofluorescence representative images of both hemispheres (contra- and ipsilateral) showing C/EBPβ expression (green) together with Iba1, a microglial marker (red). Nuclei were stained with DAPI. Insets show high magnification of the selected area. Scale bar = 100 μm. Insets scale bar = 25 μm.

Once of the events that takes place in PD together with the loss of dopaminergic neurons is the sequential hyperactivation of the surrounding glial cells in humans^31^ and animal models^32,33^. Because 6-OHDA injury in the *SNpc* results in the activation of the glial cells in this area of the brain, we next analyzed the expression of GFAP, as a marker of astroglial cells (Fig. 5), and Iba1 (Fig. 6) to label microglial cells, 7 and 15 days after 6-OHDA intracranial injection.

**Figure 7:**
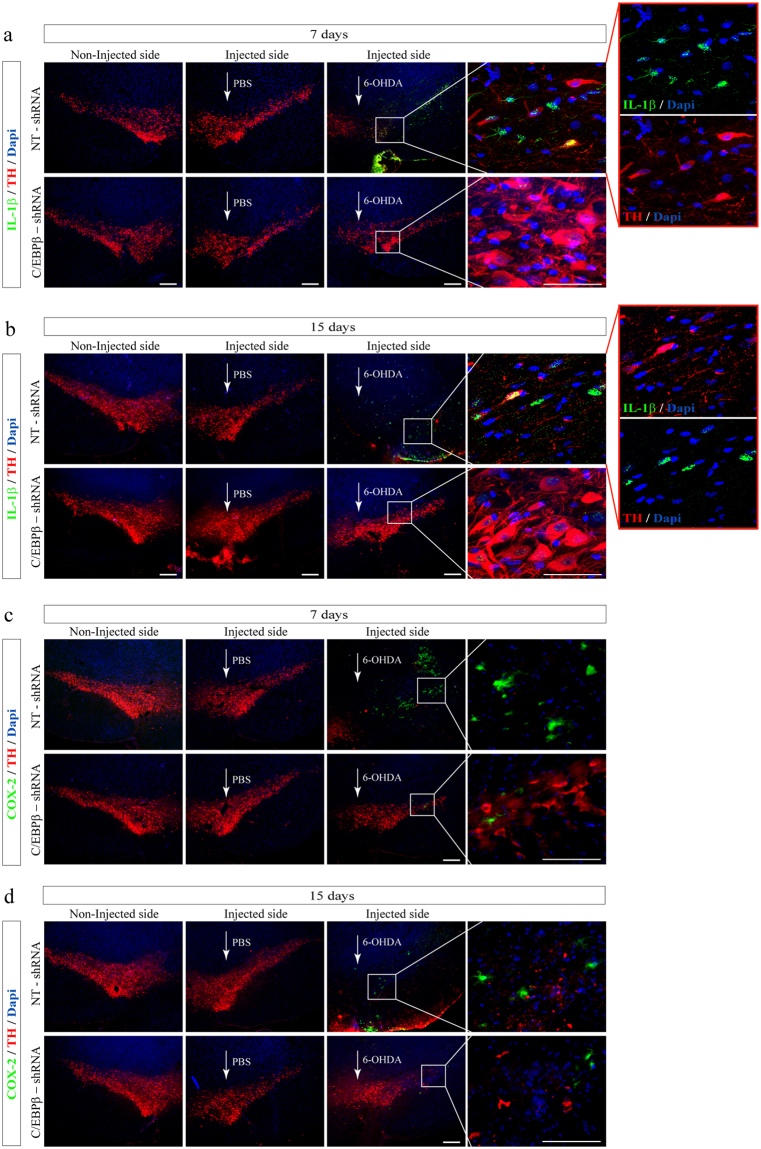
Injection of lentiviral particles containing C/EBPβ shRNA in the Substantia nigra pars compacta of adult rats significantly reduces pro-inflammatory factors production in a *in vivo* model of Parkinson’s disease. Rats were injected into the SNpc with lentiviral particles containing Non-Targeting shRNA (NT-shRNA) or C/EBPβ-shRNA in combination or not with 6-OHDA (5 μg). After 7 (**a**,**c**) and 15 (**b**,**d**) days animals were sacrificed, and the brains processed for immunofluorescence, as indicated in Methods. Double immunofluorescence representative images of both hemispheres (contra- and ipsilateral) showing pro-inflammatory interleukin 1β (IL1β) (**a**,**b**) or ciclooxigenase 2 (COX-2) (**c**,**d**) expression (green) together with the dopaminergic marker tyrosine hydroxylase (TH, red). Nuclei were stained with DAPI. Insets show high magnification of the selected area. Scale bar = 100 μm. Scale bar (insets in a and b) = 50 μm.

Evaluation of astrogliosis revealed the accumulation of GFAP-positive astrocytes within the damage area of the *SNpc*, very close to the dopaminergic neurons, of lesioned NT- shRNA (control) animals (Fig. 5), 7 and 15 after lesion. When C/EBPβ expression was analyzed inside this area, high expression of this protein was found associated with reactive astroglial cells (as seen by the changes in morphology and GFAP intensity) expressing GFAP, at both times studied (Fig. 5). The increase in astrogliosis was not observed after C/EBPβ silencing, and the pattern of GFAP immunostaining in these animals was indistinguishable from that of controls (PBS-injected).

Concerning microglial cells, identified as Iba1-positive cells (Fig. 6), fluorescence analysis revealed a considerably larger response in control (non-targeting shRNA) animals 7 (Fig. 6a) and 15 (Fig. 6b) days after 6-OHDA injection in comparison with those injected with saline. Those animals exhibited a strong microglial activation evidenced by the strong fluorescence signal found surrounding the lesioned area. As depicted in Figs 6 and 7 and 15 days after 6-OHDA injections microglia in the lesioned SN displayed reduced branching behavior, increased cell size and a stronger Iba1-immunoreactivity that is upregulated in reactive microglia in the 6-OHDA rodent model of PD^34^. These microglia morphology changes have been described as reactive changes in several CNS disease models^35^ and are used as a crude readout for microglia activation. As observed with astroglial cells, higher level of C/EBPβ was found co-localizing with activated microglial cells in these animals at all times studied (Fig. 6). By contrast, the number of reactive microglial cells in C/EBPβ silenced animals at 7 and 15 days after injury was practically absent in comparison to that found in 6-OHDA-lesioned animals infected with NT-shRNA (controls). This microglia was similar to those found on control sides, showing ramified morphologies with fine processes as demonstrated using Iba1-immunohistochemistry. These results indicated that microgliosis was completely abrogated in C/EBPβ silenced animals.

Altogether, the data above reinforced our hypothesis that targeting C/EBPβ can ameliorate PD progression not only by directly preserving dopaminergic cells, but also diminishing the subsequent harmful inflammatory process.

Then, we analyzed the production of two well-known pro-inflammatory factors implicated in neuroinflammation, IL-1β and COX-2 (Fig. 7). The levels of the two pro-inflammatory agents were elevated in the *SNpc* of control (NT-shRNA) animals as a consequence of intracerebral injection of 6-OHDA after 7 (Fig. 7a,c) and 15 (Fig. 7b,d) days. The increase in these agents is mainly observed in those areas characterized by a dramatic loss of dopaminergic cells, as detected by the lack of tyrosine-hydroxylase expression. Again and, in accordance with the lack of glial activation, in rats injected with C/EBPβ-shRNA together with 6-OHDA no upregulation of IL1β or COX-2 was observed. These results are in agreement with those obtained *in vitro* in FC1 cells treated with 6-OHDA, compared to the expression detected in control FS cultures.

### Intranigral delivery of C/EBPβ lentivirus particles significantly reduced 6-OHDA-induction of α-synuclein levels in hemiparkinsonian rats

**Figure 8:**
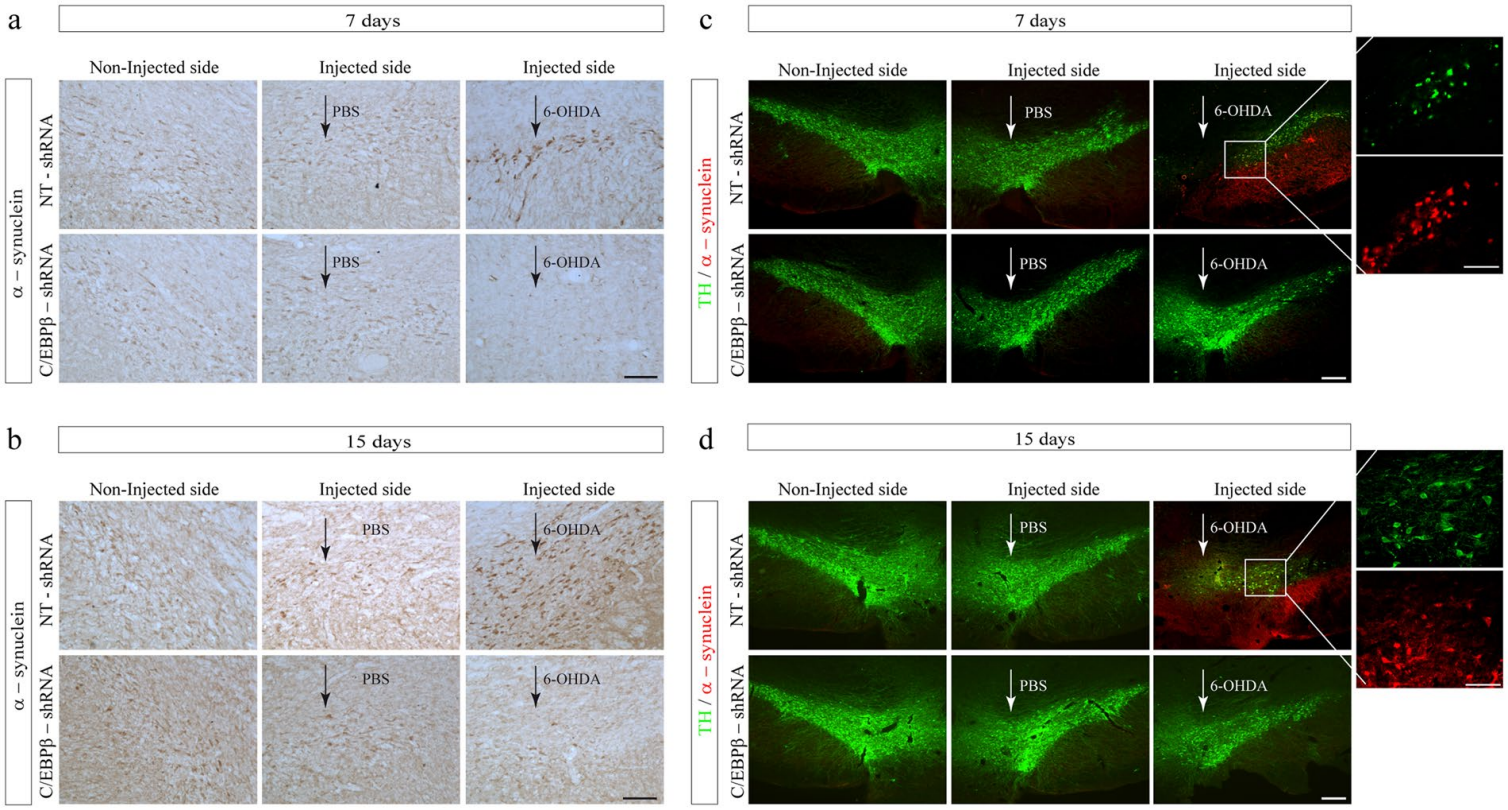
Injection of lentiviral particles containing C/EBPβ shRNA in the Substantia nigra pars compacta of adult rats significantly reduces the expression of α-synuclein in a *in vivo* model of Parkinson’s disease. Rats were injected into the SNpc with lentiviral particles containing Non-Targeting shRNA (NT-shRNA) or C/EBPβ-shRNA in combination or not with 6-OHDA (5 μg). After 7 (**a**,**c**) and 15 (**b**,**d**) days animals were sacrificed, and the brains processed for as indicated in Methods. (**a**,**b**) Immunohistochemistry of coronal sections containing de SNpc showing the expression of α-synuclein after 6-OHDA-induced damage at indicated times. (**c**,**d**) Double immunofluorescence representative images of both hemispheres (contra- and ipsilateral) showing the dopaminergic marker tyrosine hydroxylase (TH, green) together with α-synuclein (red). Nuclei were stained with DAPI. Insets show high magnification of the selected area. Scale bar = 100 μm.

Finally we studied whether the depletion of C/EBPβ could affect α-synuclein accumulation. This protein is known to increase in response to 6-OHDA treatment of dopaminergic cells *in vitro* ^36,37^. As expected, after 6-OHDA-injection, an increase in the expression of the α-synuclein protein was observed in the *SNpc* of NT-shRNA animals, 7 and 15 days after damage in comparison with saline-injected animals (Fig. 8a,b). The induction of α-synuclein levels after 6-OHDA injection was diminished when C/EBPβ gene expression was silenced, as can be observed by the diminution of immunoreactivity in the *SNpc* of these animals. To determine whether these changes in α-synuclein took place in dopaminergic neurons, we performed double immunofluorescence analysis (Fig. 8c,d). As shown in these images, α-synuclein was indeed expressed in dopaminergic neurons. In these cells, the intracellular distribution of the immunoreactivity was predominantly cytoplasmic, which is consistent with the known subcellular localization of this protein.

## Discussion

Neurodegenerative diseases represent a major medical, societal, and economic burden and are a leading cause of disability worldwide. PD is the second most common neurodegenerative disorder affecting 2–3% of the population ≥65 years of age. Different mechanisms have been implicated in PD pathogenesis, including oxidative stress, neuroinflammation, protein aggregation, mitochondrial dysfunction, excitotoxicity, etc.

Many efforts have been focused in finding new therapeutic treatments and also those genes implicated in the onset and development of PD. In fact several genes, including α-synuclein, leucine rich repeat kinase 2, and glucocerebrosidase have been found to be involved in genetic predisposition. However, much work is still needed in order to elucidate the basic mechanisms and molecular underpinnings of this disease.

A better understanding of what drives PD would help to find new effective therapeutic treatments that could delay the progression of the disease. The present study shows for the first time that shRNA-mediated depletion of C/EBPβ (a gene involved in inflammation and excitotoxicity) resulted in a neuroprotection of dopaminergic cells and attenuates glial activation in a well-established model of PD (stereotaxic injection of 6-OHDA into the *SNpc*). The data further confirmed the essential role of C/EBPβ in the neuronal cell loss and glial activation that occur after a brain injury^17,22,38^ and suggest that inhibition of C/EBPβ expression or activity may be useful in protecting the nigrostriatal dopaminergic system in PD. In this regard we have previously reported that C/EBPβ regulates the expression of genes involved in inflammatory processes and brain injury^17,18,38^. Additionally, the knockdown of C/EBPβ down regulates the expression of several pro-inflammatory agents, such as COX-2 and IL-1β, which can be attributed to the ability of C/EBPβ-specific shRNA to inhibit the glial activation in 6-OHDA-injected animals. These results are in agreement with previous data showing that C/EBPβ regulates the expression of various cytokine and chemokine genes, as well as other known pro-inflammatory agents, which play an important role in the neuronal cell death that occurs after a brain injury. The regulation of many of them is probably directly dependent of C/EBPβ. This is the case of IL-1β, TNFα, and COX-2, whose promoters contain consensus binding sites for this transcription factor^8,39^.

As mentioned in the Introduction, neuroinflammation could play a critical role in the onset and progression of PD. Although there are some discrepancies in epidemiological studies, there is one work showing that users of ibuprofen presented a significant lower risk to develop PD, compared to control non-users^40^. The results presented here clearly show that depletion of C/EBPβ results in a significant reduction in glial activation followed by a decline in pro-inflammatory agents. Thus, our data suggest that the observed effects of C/EBPβ interference on dopaminergic cell neurodegeneration could be mainly due to its regulation of inflammation and liberation of pro-inflammatory agents. Furthermore, it has been shown that chronic oral administration of different toxins, such as rotenone^41^ or 6-OHDA treatment of SH-SY5Y cells^36,37^ induces cytoplasmic accumulation of α-synuclein in surviving dopaminergic neurons and that this accumulation is a strong inducer of neuro-inflammation. The results presented in this work also show that the increased levels of α-synuclein in response to 6-OHDA injection are clearly reduced in those animals depleted of C/EBPβ, reinforcing our hypothesis that regulation of dopaminergic cell death by this transcription factor could indeed be due to its modulation of neuro-inflammation.

In conclusion, we show here that targeted delivery of C/EBPβ shRNA molecules expressed from lentiviral vectors in the *SNpc* is a useful strategy for dopaminergic protection, thus this could be the basis for future gene therapeutic strategies using secure viral vectors. Gene therapy is a promising tool for the treatment of neurological disorders due to their ability to infect both dividing and non-dividing cells. Several groups have conducted numerous preclinical and clinical studies using recombinant viral vectors, such as adeno-associated virus and lentivirus to treat these disorders, including PD^42^. Although previous studies have suggested that C/EBPβ could be involved in neurodegenerative diseases, this is the first study to show a direct relationship between C/EBPβ expression levels and PD pathological progression.

## Methods

### Animals

Adult male Wistar rats obtained from the Animal Resource Facility at the Instituto de Investigaciones Biomédicas (CSIC-UAM) were housed and maintained on food and water ad libitum in a 12 h dark–light cycle. All experimental procedures were specifically approved by the “Ethics Committee for Animal Experimentation” of the Instituto de Investigaciones Biomedicas and carried out in accordance with the European Communities Council, directive 2010/63/EEC and National regulations, normative 53/2013, on the use and care of laboratory animals. Special care was taken to minimize animal suffering.

### SH-SY5Y human dopaminergic cell line

A fast-growing human dopaminergic SH-SY5Y cells were used in this work. Cells were propagated and maintained in a medium containing DMEM supplemented with 10% fetal bovine serum, 10 mM L-Glutamine, and 5 mg/mL penicillin/streptomycin (Invitrogen). The cells were cultured in a cell incubator (ShelLab) at 37°С, 90% humidity, and 5% CO_2_. The medium was replaced by a new one every 3 days. The cells were passaged every 6 days depending on the growth rate.

### Embryonic mesencephalic cell cultures

Cultures were derived from the ventral mesencephalon (VM) of rat embryos at embryonic day 14 were established as previously described^43^. Briefly, ventral mesencephalon was isolated in cold Hanks’ balanced salt solution medium Ca2+ and Mg2+ free, washed several times, and tissue digested 15 minutes at 37 °C in trypsin-EDTA plus DNase (0.05%). VM was then gently minced and triturated with a micropipette, and the supernatant was collected and centrifuged at 1,200 g for 5 minutes. The pellet was resuspended in DMEM/Ham’s-F12 (1:1) containing 0.5 mM glutamine, 200 U/ml penicillin, and 200 μg/ml streptomycin (Gibco, Grand Island), 1% fungizone, 10 ng/ml epidermal growth factor (EGF; PeproTech), 10 ng/ml fibroblast growth factor (FGF; PeproTech), and 1 × B27 (Gibco). The cells were seeded onto 12-well plates (∼40,000 cells per cm^2^).

### Viral infection of SH-SY5Y cells

Human C/EBPβ expression was silenced in SH-SY5Y cell cultures using the FUGW lentiviral vector, as previously described^44^. The interfering selected sequence was: 5′-GAA GAC CGT GGA CAA GCA C-3′ (pool FC1). A Non-Targeting (NT) sequence (5′-GCC GCT TTG TAG GAT AGA G-3′, pool FS) was used as control. These lentiviral vectors were obtained from Dr. Quintanilla-Martinez. For lentiviral infections, 293 T cells were transiently transfected with the appropriate lentiviral expression vector and the vectors pMD2-G, pMDLg/pRRE, and pRSV-Rev, which encode lentiviral proteins. The medium containing lentiviruses was recovered, filtered through a 0.45-μm filter and added to the recipient cells. The same procedure was repeated 8 and 24 hours later. Pools FS (expressing a non-targeting shRNA) and FC1 (expressing a shRNA against C/EBPβ) were used in this study.

### Total RNA extraction and Quantitative Real Time-PCR

Total RNA was purified from SH-SY5Y culture cells after specific treatment using TRI reagent (Sigma, Saint Louis, Missouri, USA), following manufacturer´s protocol. One µg of total RNA were used for the synthesis of cDNA with the RETRoscript kit (Ambion, Austin, Tx) using pd(N)6 random hexamer as primers. Q-PCR was performed using TaqMan Gene Expression Assays and the TaqMan Universal Master Mix II Mix according to the manufacturer’s instructions (Applied Biosystems, Warrington, UK). Assay IDs of gen CEBPß was Hs00942496-s1. Amplification was conducted on duplicate samples using the ABI 7900 Detection System. Amplification of the 18 S rRNA was used as an endogenous control to normalize all assays. The relative mRNA content was determined with the 2-ΔΔCt method^45^.

### Immunoblot analysis

Total proteins were isolated from cell cultures using ice-cold RIPA buffer (0.5% sodium deoxycholate, 0.1% SDS, 1% NP-40, 1xPBS, containing protease and phosphatase inhibitors. A total amount of 30 µg of protein was separated on a 10% SDS-PAGE gel and transferred into nitrocellulose membranes (Protran, Whatman). The membranes were blocked in Tris-buffered saline with 0.05% Tween-20 and 5% skimmed milk, incubated with primary and secondary antibodies, and washed according to standard procedures. The following antibodies were used: mouse monoclonal anti-C/EBPβ (A16 clone, Abcam) and mouse anti-α-tubulin (Sigma) and mouse anti-GAPDH (Millipore). Secondary peroxidase-conjugated rabbit anti-mouse antibody (Jackson Immunoresearch) was used. Representative images of at least three independent experiments corresponding to three different samples are shown. Quantification analysis was performed using the Scion Image software. Values in the text are the mean of at least three different experiments.

### Immunocytochemistry

Fluorescence immunocytochemical analysis on cell cultures were performed as previously described^43^. Briefly, at the end of the treatment period with 6-OHDA (35 µM), SH-SY5Y or primary mesencephalic cultures, grown on glass cover-slips in 24-well cell culture plates were incubated at 37 °C for 1 h with primary antibodies directed against C/EBPβ (mouse; Abcam), Cd11b (mouse; Serotec), Cyclooxygenase-2 (rabbit; Cayman), Interleukin-1β (rabbit; Abcam), GFAP (mouse; Sigma) and tyrosine-hydroxylase (rabbit; Millipore). After several rinses in PBS, samples were then incubated with Alexa-488 goat anti-mouse, Alexa-647 goat anti-mouse and Alexa-647 goat anti-rabbit antibodies (Molecular Probes) for 45 min at 37 °C. Staining of nuclei was performed using 4′,6-Diamidino-2-phenylindole (DAPI). Images were acquired in a LSM710 laser scanning spectral confocal microscope (Zeiss). Confocal microscope settings were adjusted to produce the optimum signal-to-noise ratio. A quantitative analysis of C/EBPβ expressing cells given a particular marker (TH, GFAP or Cd11b) was undertaken using the image analySIS software (Soft Imaging System, Münster, Germany). Areas to be counted were traced at high power (400X), and at least five different counting fields were selected at random from four to eight independent experiments of parallel cultures as previously described^46^. Results are expressed as a percentage of total dopaminergic (TH positive cells), astroglial (GFAP-positive cells), or microglial (Cd11b-positive cells) co-expressing C/EBPβ.

### Nitrites measurement

Accumulation of nitrites in media was assayed by the standard Griess reaction. After stimulation of cells with 6-OHDA (35 µM) for 18 h, supernatants were collected and mixed with an equal volume of Griess reagent (Sigma-Aldrich). Samples were then incubated at room temperature for 15 minutes and absorbance read using a plate reader at 492/540 nm.

### Intracerebral 6-OHDA injections

Adult male rats (n = 5 per group) of initial body weight between 250–300 g were used. Procedures were conducted as previously described^47^. In brief, animals were properly anaesthetized and positioned in a stereotaxic apparatus (Kopf Instruments, CA). A freshly prepared solution of 6-OHDA (9 μg in 2.5 μl PBS supplemented with 0.05% of ascorbic acid) was delivered unilaterally into the left *Substantia nigra* at a speed of 1 μl/minute. The stereotaxic coordinates according to the atlas of Paxinos and Watson^48^ were as follow (from Bregma): A/P:−4.8 mm; L: +2.0 mm; D/V: −8.2 mm. Control rats were treated in the same manner but received equivalent volume of saline. Rats were then housed to recover and sacrificed at 3, 7, and 30 days after lesioning.

### Construction of shRNAs and injection of lentiviral particles *in vivo*

To knockdown C/EBPβ expression in rats, a target sequence against rat C/EBPβ was designed (NCBI Reference: NM_024125.5) using the following oligonucleotides: sense (5′-GAG CGA CGA GTA CAA GAT G-3′) and antisense (5′-CAT CTT GTA CTC GTC GCT CTT-3′). Annealed oligonucleotides were cloned in BamHI/EcoRI sites into pGreenPuroTM shRNA Cloning and Expression Lentivector (SBI, System Biosciences) according to the manufacturer’s protocol, in which shRNA was expressed under the control of the H1 promoter. The control pGreenPuro™ construct with the Luciferase shRNA Template provided by System Biosciences (Palo Alto, CA) was used as control. Generation and concentration of lentiviral particles were performed as previously described^49^. Animals were anaesthetized and placed in a stereotaxic apparatus as described above, and C/EBPβ-shRNA or control nontargeting shRNA lentiviral particles (10^6^ IU/animal) were injected into the right side of the *SNpc* in combination with 6-OHDA (9 μg) or saline. Rats were then housed to recover and sacrificed at 7 and 15 days after lesioning. Animals injected only with non-targeting shRNA were used as controls throughout all the *in vivo* experiments.

### Immunohistochemistry

Brains were processed as previously described^47^. Briefly, after perfusion of the animals with 4% paraformaldehyde, brains were removed, postfixed in the same solution at 4 °C overnight, cryoprotected in 30% sucrose, frozen, and finally 30-μm coronal sections were obtained using a cryostat. Brain sections were used for TUNEL, immunohistochemistry and immunofluorescence analysis. The following primary antibodies were used: rabbit polyclonal anti-tyrosine-hydroxylase (TH) (Millipore), to label dopaminergic cells, followed by a secondary Alexa-Fluor405 goat anti-rabbit (Fig. 3) or Alexa-Fluor647 goat anti-rabbit (Figs 4 and 7) or Alexa-Fluor488 goat anti-rabbit (Fig. 8); mouse monoclonal anti-C/EBPβ (Abcam) followed by a secondary Alexa-Fluor488 goat anti-mouse; mouse anti-glial fibrillary acidic protein (GFAP) (1/300; Sigma, Germany), for detection of astroglial cells, followed by a secondary Alexa-Fluor 647 goat anti mouse; microglial cells were stained with with rabbit anti-Iba1 antibody (Wako) or Texas Red (emission at 546) Lycopersicon esculentum (tomato lectin; Vector Labs USA); rabbit polyclonal anti-cyclooxygenase-2 (COX-2, Cayman) and rabbit polyclonal anti-Interleukin-1β (IL-1β, Abcam) both followed by a secondary Alexa-Fluor488 goat anti-rabbit and mouse monoclonal anti-α-synuclein (BD Transduction laboratories), followed by a secondary Alexa-Fluor647 goat anti-mouse (Fig. 8c,d) or by a secondary biotinylated-goat-anti-mouse (Fig. 8a,b). All secondary antibodies were used at 1/500. Fluorescent images were acquired using a Radiance 2100 confocal microscope (Bio-Rad, Hercules, CA). To compare fluorescence signals from different preparations, confocal microscope settings were fixed for all samples within the same analysis and adjusted to produce the optimum signal-to-noise ratio.

### Cell Count Analysis

To estimate the total numbers of dopaminergic neurons (TH-reactive cells), a modified stereological approach was used as previously described^49^. Confocal images of serial coronal sections (30 μm) containing the entire SNpc (rostrocaudal extent) were acquired under a × 63 objective to avoid oversampling errors. Every sixth section was selected to count the number of TH-positive cells, determining the boundaries of the SNpc with reference to internal anatomic landmarks^48^. Images were analyzed using computer-assisted image analysis software (Soft Imaging System Corporation, Lakewood, CO). Six rats per group were used. The results were expressed as the total number of labeled cells in the SNpc by multiplying the average number of labeled cells/ section by the total number of 30 μm thick-sections containing the SNpc.

### TUNEL assay

To examine the process of DNA fragmentation in the *SNpc*, TUNEL-staining was performed using the DeadEnd Colorimetric Apoptosis Detection System (Promega Corporation, Madison, WI) according to the manufacturer’s instructions. Shortly, brain sections containing the *SNpc* were treated with 20 μg/ml proteinase K and incubated for 15 minutes at room temperature, washed in PBS and fixed in 4% PFA. DNA was end-labeled using biotinylated dUTP in TdT buffer at 37 °C for 60 min. After washing, 0.3% hydrogen peroxidase was used to block endogenous peroxidase. Streptavidin horseradish peroxidase (1:500 in PBS) was added for 30 min at room temperature. DNA strand breaks were visualized using hydrogen peroxidase and the stable chromogen DAB.

### Statistics analysis

Statistical comparisons for significance were performed by ANOVA using the SPSS statistical software package (version 20.0) for Windows (Chicago, IL) followed by post hoc (Bonferroni) test to analyze differences, which were considered statistically significant at p < 0.05.

### Data availability

The data that support the findings of this study are available from the corresponding author upon reasonable request.
